# Development of Emotional Intelligence through Physical Activity and Sport Practice. A Systematic Review

**DOI:** 10.3390/bs9040044

**Published:** 2019-04-24

**Authors:** José Luis Ubago-Jiménez, Gabriel González-Valero, Pilar Puertas-Molero, Inmaculada García-Martínez

**Affiliations:** 1 Department Didactics of Music, Plastic and Corporal expression, University of Granada, 18012 Granada, Spain; jlubago@ugr.es (J.L.U.-J.); ggvalero@ugr.es (G.G.-V.); pilarpuertas@correo.ugr.es (P.P.-M.); 2 Didactics and School Organization, University of Granada, 18012 Granada, Spain

**Keywords:** emotional intelligence, emotional competence, physical activity, sport, exercise

## Abstract

At present, knowledge of physical and cognitive aspects is essential in the sporting context. Faced with this situation, the control and knowledge of emotions has a person on himself and on others, affects the sporting action. The aim of this work is to examine the relationship between emotional intelligence and the practice of physical activity. Through a systematic review in databases such as the Web of Science and Scopus that contain the terms of emotional intelligence along with the parameters of physical activity and sport. Twenty-four articles comprised the sample for further analysis. By way of conclusion it can be said that the main field of study of emotional intelligence related to the practice of physical activity is educational. Likewise, emotional intelligence is a determining factor in the improvement of sports competences.

## 1. Introduction

According to Goleman [1], emotional intelligence (EI) is a person’s inherent ability to control and self-regulate one’s feelings, to know the emotions of others, and to use emotions and feeling as a beacon of one’s actions and thoughts. Bar-On [2], define EI as the ability or capacity to perceive, integrate, understand, and manage emotions that have to do with the understanding of oneself and others, and to face different demands more successfully. Other studies [3,4,5] highlight the importance of acquiring a better knowledge of emotions, exercising a control over them, having the capacity to express them in oneself, and to identify them in others. In addition, it also implies the capacity to develop self-motivation and the promotion of a positive attitude towards life.

Authors who have performed extensive research on the subject of EI [1,6,7] propose measures that characterize the end of EI. In this way, components such as self-awareness, self-regulation, or the motivational aspect that tries to generate in the person a situation where conflicts are resolved. Other elements that can be considered essential are empathy and social skills. Empathy refers to the ability to understand the emotions of others and to treat people according to their emotional reactions. On the other hand, social skills consist in the construction of relationships with others, finding a common space of coexistence for all.

In this way, in recent years there has been a considerable increase in studies related to sports practice and EI, some from the educational field related to physical education [3,8,9,10,11,12,13], others from competitive sport [14,15,16], and from sports psychology [17,18].

According to the studies consulted, it is appreciated how the practice of physical activity and sports offer many satisfactions, in exchange for many efforts. Physical activity incorporates relaxation, offering an opportunity to face challenges, as well as a way of collaboration and motivation in a team or a competition with oneself [19,20]. IE and physical activity are intimately related, to such an extent that many of the techniques of relaxation, concentration, visualization are shared in an increasing number of clubs, federations, and even coaches are hiring more professionals to implement these techniques, aiming to improve the performance of athletes [21]. In the same way, it emphasizes the importance of physical activity practice, at the time of producing improvement in the physical, psychic, and social aspect, as well as the quality of life as reflected [22]. In the field of physical practice, sports psychology is responsible for analyzing, studying, and observing the behaviors, reactions, and emotional responses of the individual or team at the time of competition [18]. For this, the individual must be able to regulate and positively control his emotions in such a way that they do not negatively influence the sporting gesture and favor quick and effective decision making [4].

Many of the tools that EI and sports practice share have the main characteristic of being tremendously practical, especially of application in daily life. The importance of an athlete being able to exercise control over his emotions in relation to sports practice is evidenced in various studies [23,24,25,26,27,28,29,30]. Hannin and Sirja [25] found that emotions in relation to sporting success present individual optimal zones, but not so at the group level. Each athlete reacts in a different way to stress by requiring a certain degree of negative or positive emotions that give him an optimal performance point in a play situation.

The aim of this study is to confirm the relationship between EI and physical activity practice through a systematic review. Indeed, we focus on the most commonly used questionnaires in each sample type. The hypothesis of the study is that high levels of EI are associated with physical activity.

## 2. Materials and Methods

### 2.1. Procedure

Our systematic review uses the guidelines of the PRISMA (Preferred Reporting Items for Systematic reviews and Meta-Analyses) statement [31], to give it greater consistency and scientific rigor. In addition, it follows the steps established in the Fernández-Ríos and Buela-Casal standards [32]. The main objective of this paper is to analyze all EI empirical studies on sport and physical activity. Two search engines were consulted: Web of Science and Scopus. This search was carried out during the months of November and December 2018, and included different combinations of the following key words, both in English and Spanish: “emotional intelligence”, “emotional competence”, “sport”, “physical activity”, and “exercise”, in addition to using the Boolean “and” and “or”. The time range for the publication of these articles was then defined, from 2008 to 2018. In this time frame, 311 articles were found, 167 in Web of Science and 80 in Scopus.

In this way the population of the present study could be fixed. The search was then refined by considering only the articles published in the research domain “Social Science” and having as a priority the research areas “Sport Sciences” and “Education Educational Research”. In order to select the study sample, the following inclusion criteria were used to identify the relevant articles: studies of an empirical nature; EI had been evaluated in the context of physical or sporting activity and research showing statistical results. Papers such as doctoral theses and communications or articles with only published abstracts were therefore excluded. Before applying the inclusion criteria, we deleted 64 articles due to duplication.

**Figure 1:**
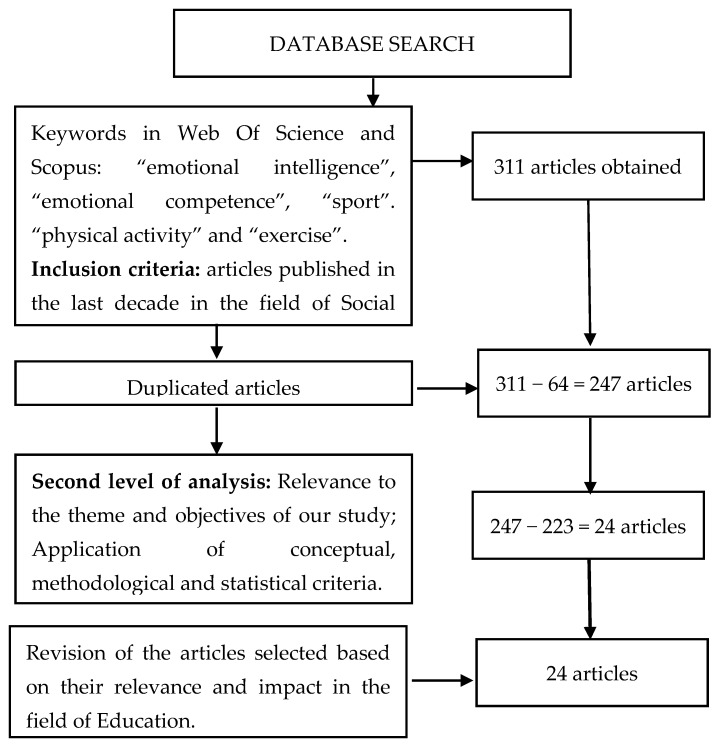
Article selection flowchart.

The application of these inclusion criteria was carried out by means of a first reading of the title and summary of the study population, consecutively, a systematic reading of the full text of the articles was made. In this way, and with the application of conceptual, methodological and statistical criteria, a total of 223 studies were eliminated (Figure 1).

In order to process the data, a logical order comparison of the data was carried out, and all the information obtained was synthesized in order to achieve a truthful and current study.

### 2.2. Population and Sample

Following the above sequence, the population of this study corresponds to 247 articles, extracted from the Web of Science (WOS) and Scopus. After the application of the inclusion criteria, the sample of this review amounts to 24 scientific articles.

## 3. Results

The 24 articles comprising the sample of this systematic review have a total sample participation of 10,395, as shown in Table 1. In order to extract the data, the following coding process has been taken into account: (1) author(s) and year; (2) study type; (3) population; (4) sample, men and women; (4) mean age and standard deviation; (5) the instruments used; and (6) main results.

The articles that constitute the corpus of the study have been developed in the stages of Primary Education, Secondary Education, University, sports, and people over than 40 years-old. In addition to future teachers, Kindergarten Education was the only stage where no scientific studies were developed.

With regard to the scope of study of the articles analyzed (Table 2), they consist of a greater number of investigations related to EI and physical activity, it is the university stage (45.83%), followed by sports disciplines (33.3%). In this way, we highlight the scarcity of studies that work in educational stages such as Primary Education, Secondary Education and over than 40 years-old (8.3%).

In addition, Table 3 shows the countries where the various researches that address training and work performance have been developed in relation to EI. Spain (N = 13) is the country that has published the most scientific productions, followed by Great Britain (N = 4) and United States (N = 2).

## 4. Discussion

In line with previous studies [33], we have been able to highlight how EI is an influential factor in sport. EI helps athletes in decision making, memory, reasoning, and problem solving. EI could also be associated with stress regulation or athletes’ own motivation to exercise.

Table 1 shows the main results obtained in each of the studies reviewed. Likewise, our hypothesis is confirmed, highlighting how in most of the investigations the practice of physical activity is related to high levels of IE. In addition, a high level of EI also reduces stress caused by a sporting competition.

In the first part we analyzed how the TMMS-24 questionnaire is the most used in the educational field and consists of three key dimensions of EI with eight items each, measuring emotional perception, understanding of feelings, and emotional regulation. Similar results were obtained in [34,35], differences in EI between gender are very similar, significant differences were found in the attention to feelings in which women present a higher score than men. Other investigations [36,37,38] coincide in their findings regarding the practice of physical activity. This produces favorable changes in the three components of EI: perception, understanding, and regulation of emotions. The TEIQue test focuses on detecting how people understand and manage their emotions, how they interpret and manage the emotions of others, and how they use this knowledge to manage their relationships. Other studies [7,34,35,36,37,38,39,40] highlight how this questionnaire makes it possible to measure EI in sportspeople. At the same time, they identify findings on the positive repercussion that the practice of physical and sports exercise has on the control and detection of one’s own and other people’s emotions.

The objective of the WLEIS scale is to evaluate the level of IE perceived, covering four factors such as the evaluation of one’s own emotions or intrapersonal perception; the evaluation of the emotions of others or interpersonal perception; the use of emotions or assimilation; and the regulation of emotions. The study in Reference [41] demonstrates that EI functions as a protective factor of physical health by enhancing the practice of physical activity. The authors point out how emotionally more intelligent individuals are more motivated to engage in regular physical activity because of the opportunities for social interaction with others that the activity itself offers, making significant differences in anger, calm, confusion, fatigue, happiness, and strength in repeated episodes of intense exercise [42,43]. In this way, they maintain that high levels of EI are associated with emotions pleasant by the practice of physical exercise obtaining higher levels of calm and happiness along with a decrease in anger, depression and fatigue. Similarly, and with a sample of coaches, the study conducted in Reference [44] obtained results in which they reflect how the totality of their sample ensures that they are able to evaluate and regulate their personal emotions, paying attention and detecting the emotions of their athletes.

Another study [45] used the BarOn EQ-I questionnaire to identify various mechanisms to detect the influence of sports practice and its effects on the emotions of participants, including fitness or weight loss increased endorphin production after exercise, producing substantially positive changes in self-esteem controlling the emotions themselves to develop motor skills. They also establish a positive relationship in terms of increased physical activity and being emotionally healthy. Lu, Li, Hsu, and Williams [46] investigate how stress management, intrapersonal emotions, helps athletes cope effectively with adversity. Therefore, stress management appears to be an important factor in assessing athletes’ emotional competence [47]. These results further emphasize the positive impact of physical exercise on EI, and the study in Reference [48] obtained significant differences among non-athletes.

The GES scale [49,50] was used at university level without finding significant gender differences. The study developed by Duran et al. [51] identified difficulties in identifying emotions. On the other hand, the ease with which emotional awareness can be identified after the practice of physical activity is highlighted. Other investigations [15,52,53] agree on the finding regarding the differences in IE between individual and collective sportsmen and women, with the exception of those who do not practice physical activity [54].

## 5. Conclusions

Following a comprehensive systematic review, the number of articles studying the link between EI and the practice of physical activity and sport has increased considerably since 2013. At the same time, it can also be appreciated that practically all of the studies reviewed are cross-sectional, only one study was of longitudinal type. This infers the pressing need for more implementations and interventions, due to the results reported on its participants.

In relation to the country in which the different studies have been carried out, we find a total of 10 different countries. Spain stands out, over the rest, with 13 investigations; Great Britain with four; USA and Taiwan with two each; while Colombia, Costa Rica, France, Hungary, Iran and Italy have only had one study in the last ten years. 

Most of the documents focus on the study in the university stage (11 articles), this may well be due to the rapid access to the university population that the researchers have. The study of EI in different sports disciplines achieves a total of 8 articles in the last ten years, highlighting the study and relationship of EI in athletes. Continuing with the field of study, there is research in the stages of Primary and Secondary education, two articles each, and in a population of more than 40 years, with two other articles.

In conclusion, it could be said that more than half of the research reviewed in this work has as its thematic axis the EI study related to the practice of physical activity and sport in an educational environment. This fact makes us stress the need for studies in groups outside educational practice, always bearing in mind that most of the existing ones are of a transversal nature. Perhaps, the access to a wide sample is the great handicap for the study in other sport fields.

## Figures and Tables

**Table 1:** Data from the systematic review studies.

Author and Year	Study Type	Population	Sample	Years M/SD	Instrument	Main Results
			M/W			
Laborde et al. 2017	T	Athletes	972 503/469	21.16 ± 4.8	TEIQue	Positive relationship between trait EI and physical activity goals.
Ladino et al. 2016	T	University	25	22.2 ± 3.6	TMMS-24	There are no differences after the intervention.
Cera et al. 2015	T	Secondary	170 95/75	13.24 ± 0.93	TMMS-24/BPNES	Emotional clarity relates to intrinsic motivation in sport.
Duran et al. 2015	T	Secondary	220 101/119	14.3 ± 1.93	GES	Identifies positive and negative emotions associated with game types.
Fernández-Ozcorta et al. 2015	T	University	1008 573/435	21.18 ± 2.68	TMMS-24	Higher level of physical activity is associated with higher levels of clarity and emotional repair.
Lavega et al. 2015	T	University	99 80/19	20.68 ± 2.67	GES	The practice of physical activity influences the knowledge of one’s own emotions.
Laborde et al. 2015	T	Athletes non athletes	1950 979/971	22.49 ± 1.1	TEIQue	Positive relationship between EI and resilience and optimism.
Bhochhibhoya et al. 2014	T	University	438 103/335	20.1 ± 2.38	SSEIT	Individuals who engage in physical activity are healthy emotionally.
Abad-Robles et al. 2014	T	More than 40	35	-	TMMS-24	The practice of biodanza has a positive relationship with the EI development.
Laborde et al. 2014	T	Athletes	973 519/454	21.4 ± 3.9	TEIQue	The promotion of EI reduces the stress and pressure of training and competitions.
Lavega et al. 2014	T	University	309 241/71	19.6 ± 2.32	GES	Women have more positive emotions than men in the practice of physical activity.
Saies et al. 2014	T	Athletes	347	-	CIEPDEC	Athletes under a high level of stress have greater emotional regulation in competition.
Sánchez-Gutiérrez et al. 2014	T	University	236 138/98	20.19 ± 2.47	TMMS-24	Men pay more attention to feelings and women to repairing moods.
Pulido-Martos et al. 2014	T	More than 40	115 60/55	-	WLEIS	Positive relationship between dimensions Perception of the emotions of others and Use of emotions with the physical activity practice.
García-Coll et al. 2013	T	Athletes	2091 1519/572	20.8 ± 6.14	SSRI	Adaptation of the SSRI questionnaire to sport.
Rodríguez-Peláez et al. 2013	T	University	-	-	TMMS-24 TEIQue	EI facilitates the practice of physical activity.
Ruiz et al. 2013	L	Primary	25 12/13	-	Sesions	Stress is reduced through EI in physical education classes.
Martín de Benito et al. 2012	T	Primary	117	8.9 ± 0.25	SSRI	Relationship between IE and self-determined motivation.
Bostani et al. 2011	T	University Athletes	200	25.53 ± 1.53	BarOn EQ-I SCL- 90-R	There are differences between athletes and non-athletes in the dimension’s happiness, stress tolerance and self-affirmation.
Lane et al. 2011	T	Athletes	34 24/8	-	WLEIS	Runners present significant changes in their emotions during repeated long-distance runs.
Lane et al. 2010	T	University	284 154/130	21.02 ± 2.46	WLEIS	IE relates to vigor, happiness, calmness even when the results are not as expected.
Lu et al. 2010	T	University	111 64/47	21.0 ± 2.3	BarOn EQ-I	Low IE is associated with high precompetitive anxiety.
Li et al. 2009	T	University	599 138/461	-	BarOn EQ-I	The greater the physical activity, greater EI score and stress management, general mood and adaptability.

* TEIQue: Trait Emotional Intelligence Questionnaire; TMMS-24: Trait-Meta Mood Scale; BPNES: Basic Psychological Needs Measurement Scale; PMCSQ-2: Perceived Motivational Climate in Sport Questionnaire; GES: Games and Emotions Scale; SSEIT: Schutte Self-Report Emotional Intelligence Test; CIEPDEC: Emotional Intelligence Perceived in Sports and Competitive Contexts Questionnaire; WLEIS: Wong-Law Emotional Intelligence Scale; SSRI: Schutte Self Report Inventory; BarOn EQ-I: BarOn Emotional Quotient Inventory; SCL-90-R: Symptom Checklist- 90- Revised

**Table 2:** Study number by population.

Population	Percentage
Sports disciplines	33.3% (n = 8)
Primary Education	8.3% (n = 2)
Secondary Education	8.3% (n = 2)
University	45.83% (n = 11)
More than 40	8.3% (n = 2)
Total	100% (n = 25)

**Table 3:** Study number by country.

Country	Percentage
Colombia	3.7% (n = 1)
Costa Rica	3.7% (n = 1)
France	3.7% (n = 1)
Great Britain	14.8% (n = 4)
Hungary	3.7% (n = 1)
Iran	3.7% (n = 1)
Italy	3.7% (n = 1)
Spain	48.1% (n = 13)
Taiwan	7.4% (n = 2)
United States	7.4% (n = 2)
Total	100% (n = 27)
